# A combined DFT-MD study on the adsorption behavior of allicin on silver-organic framework surfaces

**DOI:** 10.1038/s41598-025-33945-0

**Published:** 2026-01-02

**Authors:** Tareq Nafea Alharby, Muteb Alanazi

**Affiliations:** Department of Clinical Pharmacy, College of Pharmacy, University of Ha’il, Ha’il, 81442 Saudi Arabia

**Keywords:** Allicin, Ag-MOF, Adsorption behaviour, Optical absorption, DFT, Chemistry, Materials science

## Abstract

This research explores the adsorption characteristics of allicin (ALC) molecules on the metal-organic framework (MOF) designated as AgH_8_C_6_N_3_O_2_, or Ag-MOF, focusing on three different surface orientations: 100, 010, and 001. The binding strength of ALC molecules on different facets of the Ag-MOF was systematically evaluated by using density functional theory with D3 dispersion corrections (DFT-D3) and molecular dynamics (MD) simulations. Results revealed that on the (001) surface, the molecule labelled as configuration D (001-D) demonstrated a binding energy (E_bin_) of -0.894 eV. This value reflects a significantly stronger binding affinity relative to other configurations, with configuration A (001-A) showing − 0.697 eV, configuration B (001-B) at -0.732 eV, and configuration C (001-C) measuring − 0.816 eV. This stronger interaction, assessed via the PBE-D3 functional, suggests that ALC (particularly 001-D) preferentially binds more robustly to the (001) facet through physisorption, whereas interactions on the (010) and (100) surfaces are comparatively weaker in adsorption process. Significant variations in the dipole moment were observed upon ALC adsorption onto the (001) surface, which could enhance its solubility. We used Time-Dependent Density Functional Theory (TDDFT) to analyze UV-Vis spectra, finding a modest red shift in the absorption peak when ALC was adsorbed onto the (001) surface, compared to the spectrum of the pristine MOF surface. Infrared (IR) spectra calculations for both ALC and the ALC-adsorbed MOF closely matched experimental data, validating the computational approaches. MD simulations, spanning 20 to 80 picoseconds, demonstrated that ALC interacts effectively with the porous surface of the MOF, highlighting its suitability for adsorption. The theoretical analysis revealed that the electronic structure of the Ag-MOF changes when ALC molecules are adsorbed onto it. These findings offer valuable insights into the properties of ALC when adsorbed onto Ag-MOFs, providing a foundation for further exploration of their role in biomedical applications.

## Introduction

In recent years, there has been growing interest in the development of green metal-organic frameworks (MOFs) for biomedical applications^1^. Green MOFs are known for their inherent stability and acceptable biocompatibility^2^. However, their stability in water and at elevated temperatures is limited, which hinders their potential as carriers for drug delivery systems (DDS)^2^. The global concern surrounding the SARS-CoV-2 virus and the COVID-19 pandemic has prompted the development of vaccines by various organizations^3^. MOFs offer promise not only due to their physicochemical structure and morphology but also because of their ability to provide a wide range of surface interactions and porous channels. Additionally, MOFs are highly stable and well-organized 3D nanostructures with tunable pore structures through synthetic pathway manipulation^4^. By adjusting synthesis parameters such as temperature, pH, stirring speed, solvent, and precursor purity, regular or stimuli-responsive MOFs can be tailored for targeted DDS applications in different cell lines, organs, and target tissues^5^. The long-term efficacy and potential antimicrobial properties of MOFs provide alternative opportunities in the field of biomedicine^6^. MOFs themselves can exhibit antimicrobial features or serve as platforms for delivering antimicrobial agents^6^. Recent studies have explored the use of metal-organic frameworks, such as Hf-UiO-66 and Zr-UiO-66, for enhanced adsorption of curcumin and bisphenol A, respectively^7,8^.

Furthermore, allicin or diallyl thiosulfinate (ALC), the main active compound in garlic (Allium sativum), has been extensively studied for its wide range of pharmacological activities, including antimicrobial, antioxidant, anticarcinogenic, and antineuroinflammatory properties^9–12^. Researchers have also investigated the incorporation of allicin into various composites and nanoparticles to enhance antimicrobial activity and therapeutic efficacy^13,14^. Barbara E. Souza and co-authors have shown exploring 5-fluorouracil delivery mechanisms from Metal − Organic Framework nanocomposites using Time-Resolved IR Spectra and DFT calculations^15^. Mostafavi et al. investigated the interaction between glycine and metal organic frameworks (MOFs) using dispersion-corrected DFT calculations. Their findings reveal strong interactions between glycine and MOF-5, indicating the formation of chemical bonds. The accuracy of their method was validated through experimental data and second-order Møller-Plesset perturbation theory. Additionally, the study highlights a similar strong interaction between a tripeptide and MOF-5, resembling interactions observed in biological systems^16^. Safarpour and colleagues designed a novel nanocomposite by functionalizing carboxymethyl starch/metal-organic framework with folic acid, resulting in the CMS/Ag@MOF-FA material. Various characterization techniques verified the successful synthesis of this composite, which displayed a combination of rod-shaped and spherical nanoparticles. This nanocomposite demonstrated a high capacity for loading the chemotherapy drug doxorubicin, achieving an loading efficiency of nearly 83%. Additionally, it showed a pH-dependent release profile, releasing the drug more rapidly in acidic conditions (pH 5.0) than at physiological pH (7.4). When tested against HeLa cancer cells, the nanocomposite exhibited moderate inhibitory effects, reducing cell viability by about 27%. Moreover, it possessed inherent antibacterial properties, effective against both gram-negative and gram-positive bacterial strains^17^. In this study, we explore the use of Ag-MOF substrates as novel carriers for ALC molecules, investigating their impact on the structural, optical, and electronic features. The combination of Ag-MOFs with ALC could potentially offer unique advantages for therapeutic applications. To the best of our knowledge, this is the first example of utilizing Ag-MOFs for the delivery of ALC molecules and comprehensively characterizing their effects. This study aims to deepen our understanding of MOFs and investigate the potential of Ag-MOFs as carriers for ALC in biomedical applications. Specifically, it examines interactions such as binding affinity to particular facets, charge transfer, and solubility changes, which may influence drug delivery performance. The findings suggest that Ag-MOFs could contribute to enhancing the stability and efficacy of ALC, indicating potential avenues for further exploration in biomedical applications.

## Theoretical details

In this section, we detail the theoretical approach used to explore the interaction between Ag-MOF and ALC molecules. Our methodology is grounded in a first-principles framework utilizing density functional theory with DFT-D3 dispersion corrections^18^. The DFT-D3 empirical dispersion correction by Grimme et al.^18^ was employed to accurately describe the van der Waals (vdW) interactions, which are crucial for physisorption processes and the stability of MOF structures. We employ numerical atomic orbitals as the basis set for all calculations. The exchange-correlation effects are modeled using the Perdew-Burke-Ernzerhof (PBE) functional within the generalized gradient approximation (GGA)^19^. To accurately capture atomic interactions, norm-conserving Troullier-Martins pseudopotentials are used. Extensive geometry optimizations and total energy calculations are performed to identify the most stable molecular configurations^20^. The computations are carried out using the OpenMX software package^21^, which solves the Kohn-Sham equations employing a linear combination of pseudo-atomic orbitals (LCPAO) basis sets. This approach has proven highly effective in analyzing the interactions between Ag-MOF and ALC molecules^22^. The electronic loop was considered converged when the total energy difference between successive cycles was less than 1.0 × 10^− 8^ Ha. The geometry optimization (ionic loop) was considered converged when the maximum force on any atom was less than 0.001 Ha/Bohr. All atoms were modelled using double-zeta polarized basis sets. The real-space grid employed a kinetic energy cutoff of 150 Ry to ensure computational accuracy. In our study, we modeled the slabs with three atomic layers for each surface facet, specifically (100), (010), and (001). The (100), (010), and (001) surfaces were selected as they represent the most stable low-Miller-index facets for this orthorhombic Ag-MOF, allowing us to probe interactions with different surface terminations. A vacuum gap of 20 Å was applied in the direction perpendicular to the surface to prevent any spurious interactions between periodic images. A Monkhorst-Pack k-point grid of 3 × 3 × 1 was used for the surface calculations to ensure accurate sampling of the Brillouin zone. This vacuum thickness was deliberately selected to exceed the suggested minimum of 15 Å noted in the original report, ensuring that there is no unintended interaction between the periodic images of the slabs. Additionally, certain atoms were held fixed during the optimization process to maintain structural stability. We conducted our simulations within the NVT ensemble, employing a Nosé-Hoover thermostat set to a temperature of 300 K. The MD simulations were run for a total time of 80 ps, using a time step of 1 femtosecond (fs). To find the most favorable adsorption structure of ALC on the MOF, a genetic algorithm (GA) is applied. This GA implementation is integrated within the Atomic Simulation Environment (ASE) software, specifically using the “ase.ga” module, which provides the necessary tools for optimization. By combining ASE with GA, we can efficiently set up simulations and optimize the relevant material properties. The GA itself can be executed using external Python libraries such as DEAP or PyEvolve. The GA was used to find low-energy adsorption configurations. We used a population size of 32 individuals. The operations included crossover (60% probability) and mutation (40% probability). The algorithm was stopped after 50 generations without an improvement in the fitness (lowest energy) of the population. The E_bin_ was calculated using the following formula:

1 $${{\mathrm{E}}_{{\mathrm{bin}}}}={\text{ }}{{\mathrm{E}}_{{\mathrm{ALC}} - {\mathrm{MOF}}}}--{\text{ }}\left( {{{\mathrm{E}}_{{\mathrm{MOF}}}}+{\text{ }}{{\mathrm{E}}_{{\mathrm{ALC}}}}} \right)\,+\,{{\mathrm{E}}_{{\mathrm{BSSE}}}}$$

where E_ALC-MOF_ is the total energy of the Ag-MOF complex with the adsorbed ALC molecule, E_MOF_ is the total energy of the isolated, optimized Ag-MOF substrate, E_ALC_ is the total energy of the isolated, optimized ALC molecule, and E_BSSE_ is the Basis Set Superposition Error correction. By utilizing these computational methods and calculations, we can achieve a detailed understanding of how ALC molecules adsorb onto the Ag-MOF surface, including the underlying energetic interactions. The interaction energy, incorporating the DFT-D3 correction for both the Ag-MOF (A) and ALC (B), is defined as:

2 $$\:\varDelta\:E\left(AB\right)={E}_{AB}^{(\alpha\:\cup\:\beta\:)}-{E}_{A}^{\left(\alpha\:\right)}-{E}_{B}^{\left(\beta\:\right)}$$

The counterpoise energy of complex also can be determined by:

3 $$\:{\varDelta\:E\left(AB\right)}^{CP}={E}_{AB}^{\alpha\:\cup\:\beta\:}\left(AB\right)-{E}_{A}^{\alpha\:\cup\:\beta\:}\left(A\right)-{E}_{B}^{\alpha\:\cup\:\beta\:}\left(B\right)$$

The basis set superposition error (BSSE) correction then can be calculated by:

4 $$\:{E}_{BSSE}={\varDelta\:E\left(AB\right)}^{CP}-\varDelta\:E\left(AB\right)$$

The Basis Set Superposition Error (BSSE) was corrected for using the counterpoise method, as it is essential for obtaining accurate binding energies when using finite basis sets, preventing an artificial overestimation of interaction strengths.

## Results and discussion

### Adsorption behaviour

**Fig. 1:**
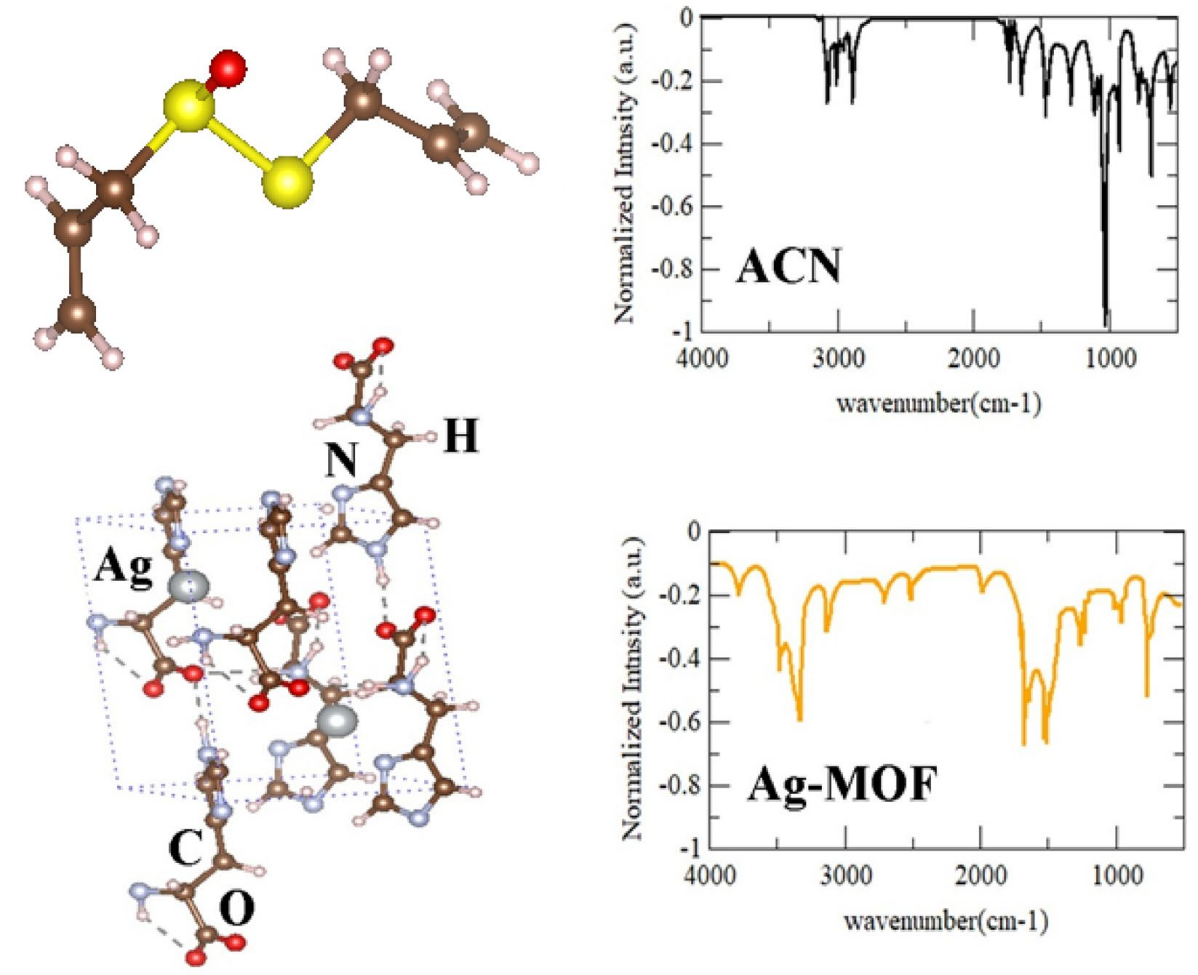
The optimized structure of ALC and Ag-MOF (0 0 1) molecules.

In order to investigate the adsorption of ALC on Ag-MOF substrates, we conducted the design and optimization of both ALC and Ag-MOF molecules, followed by the full relaxation of structures using the PBE-D3 functional (Fig. 1). In the figure, a structure of the ALC molecule is shown along with the crystal structure of MOF, in addition to the IR spectrum of the ALC molecule and MOF also are shown. Due to the presence of carbon-carbon (C-C), carbon-sulfur (C-S), sulfur-sulfur (S-S), and oxygen-sulfur (O-S) bonds in the structure of ALC molecule, the Fourier-Transform Infrared Spectroscopy (FTIR) spectrum shows well-defined peaks corresponding to the vibrations related to these bonds. The specific wavelengths associated with these bonds in the FTIR spectrum can vary depending on factors such as the exact molecular environment and experimental conditions. The theoretical infrared (IR) spectrum of ALC features prominent absorption peaks corresponding to various molecular vibrations. These include the C–S stretching vibration observed around 650 cm^− 1^, the S = O stretching mode near 1035 cm^− 1^, the C = C stretching frequency approximately at 1625 cm^− 1^, and the bending vibrations of aliphatic C–H bonds at about 1450 cm^− 1^. The experimental IR spectrum of ALC displayed the main peaks at 3423, 1655, and 1590 cm^− 1^^23^.

**Fig. 2:**
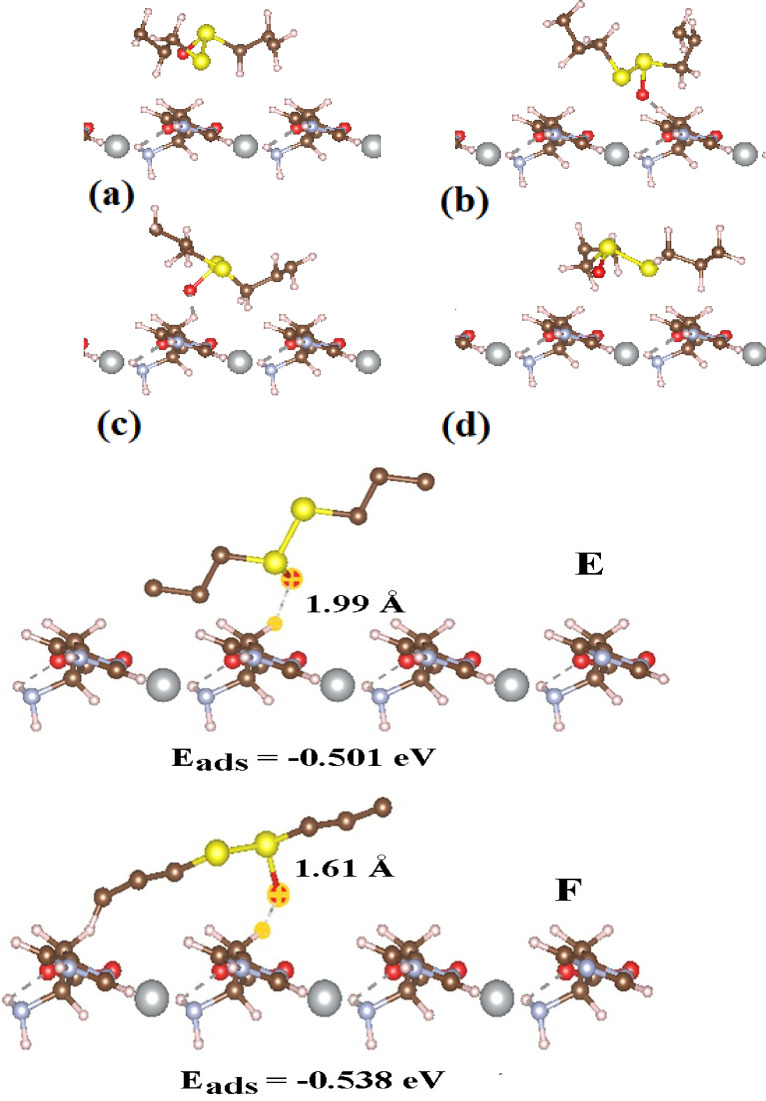
The adsorption behaviour of ALC molecule via its oxygen head onto the Ag-MOF (0 0 1) surface.

Specifically, we focused on the Ag-MOF (1 0 0), Ag-MOF (0 1 0), and Ag-MOF (0 0 1) substrates, where the sulfur and oxygen atoms of the ALC molecule interacts with the metal site, effectively closing it off (Fig. 2).

Considering the investigation of the most stable structures for ALC absorption on the 001 plane of the Ag-MOF crystal, we can analyze the interaction energy using various corrections within the realm of functionals. Table 1 provides an overview of the counterpoise and DFT-D3 correction and BSSE correction, showcasing their respective values. The configurations displayed (A-D for each surface) represent the lowest-energy structures discovered through our Genetic Algorithm (GA) exploration, indicating the most thermodynamically favorable adsorption arrangements. Examining the data in Table 1, we observe that the van der Waals energy correction ranges from − 0.69 to -0.89 eV. This correction factor accounts for the attractive forces between atoms or molecules resulting from temporary dipoles. In this case, the correction reduces the total energy of the system. Simultaneously, we note that the BSSE correction ranges from − 0.52 to -0.7 eV. BSSE stands for Basis Set Superposition Error, which addresses the overestimation of interaction energies due to the incomplete basis sets used in calculations. This correction factor aims to mitigate the overestimation, resulting in more accurate calculations. The counterpoise correction accounts for the basis set superposition error in the calculation of intermolecular interaction energies. It corrects for the spurious interaction arising from the overlapping of basis functions between different atoms or molecules. By incorporating these correction factors into the analysis, we can obtain more precise values for the interaction energies of the ALC absorption on the 001 plane of the Ag-MOF crystal. These correction factors help refine the calculations and improve the accuracy of the results, ensuring a more comprehensive understanding of the system’s stability.

**Table 1:** The DFT-D3 correction, counterpoise interaction energy, and basis set superposition error (BSSE) correction values for the analyzed structures, expressed in electron volts (eV).

Configuration	DFT-D3 correction	Counterpoise corrected interaction energy	BSSE correction
A	− 0.697	− 1.262	− 0.608
B	− 0.732	− 1.122	− 0.523
C	− 0.816	− 1.445	− 0.868
D	− 0.894	− 1.422	− 0.878

**Fig. 3:**
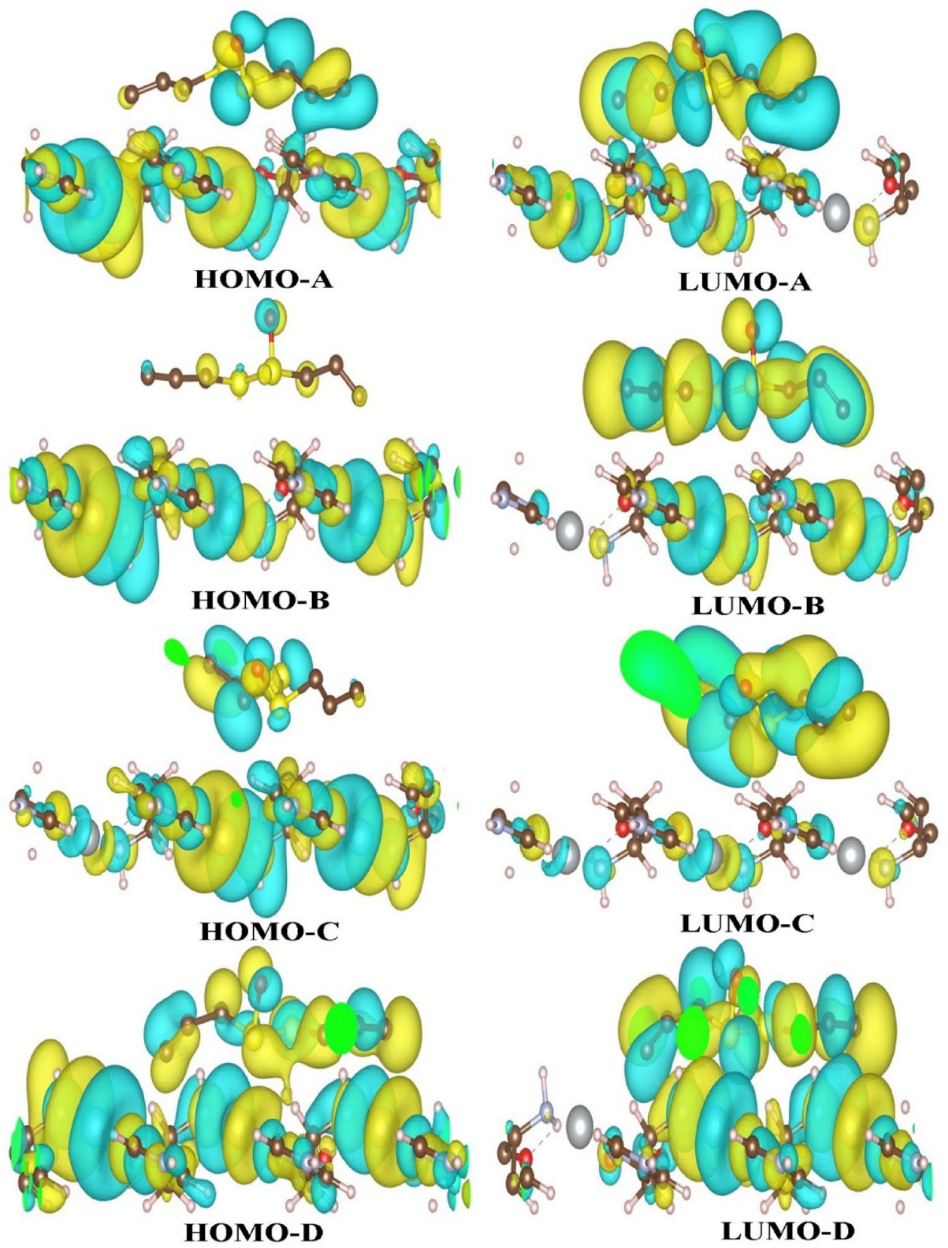
The HOMO and LUMO orbitals of ALC molecule loaded onto the Ag-MOF (0 0 1) surface.

The values of binding energy for the ALC interacting with Ag-MOF substrates are summarized in Table 2. Table 2 provides the E_bin_ values for ALC interacting with the Ag-MOF (1 0 0), Ag-MOF (0 1 0), and Ag-MOF (0 0 1) substrates by the PBE functional. Notably, when the ALC molecule contacts the Ag-MOF (0 0 1) substrate, the most stable state in 001-A exhibits an increased E_bin_ value of -0.654 eV, indicating a highly stable interaction compared to the 001-B (-0.599 eV), 001-C (-0.577 eV), and 001-D (-0.544 eV). In the subsequent study, we assessed the E_bin_ value using the PBE-D3 functional and incorporated the BSSE correction to analyze the interaction between two species. The E_bin_ values for configurations A, B, C, and D were initially determined to be -0.697, -0.732, -0.816, and − 0.894 eV, respectively. After applying the BSSE correction, these values were adjusted to -0.608, -0.523, -0.868, and − 0.878 eV, respectively. This level of binding energy is promising for drug delivery, as several studies have reported useful pharmaceutical applications with absorption energies below − 1.5 eV. The [0 0 1] surface of the Ag-MOF exhibits pores that can accommodate ALC molecules of varying configurations, with similar energies and suitable depths for accumulation. We explicitly highlight that the strong adsorption observed on the (001) surface is primarily a result of physisorption. In this process, sulfur atoms within the ALC molecule form direct coordination bonds with the silver sites inside the MOF framework. This interaction is characterized by shorter bond distances and increased charge transfer, reinforcing the electrostatic nature of the adsorption. This conclusion is supported by the shorter bond lengths, increased charge transfer, and greater binding energies associated with this interaction. In contrast, the adsorption on other surfaces is comparatively weaker and mainly governed by physical interactions, such as van der Waals forces and weak electrostatic attractions. The E_bin_ values for ALC adsorbed via its oxygen head on the Ag-MOF (0 0 1) surface were determined to be -0.501 eV in configuration 001-E and − 0.538 eV in configuration 001-F (Fig. 2). We explicitly clarify that all interactions occurring on the (100) and (010) surfaces are definitively characterized as physisorption, predominantly governed by van der Waals forces. In Fig. 3, the HOMO and LUMO wave functions for four configurations with the most stability in the interaction of ALC molecule with the MOF (001) surface are shown. As it is clear in the figure, the HOMO and LUMO wave functions that show the chemically active points, as well as the reactivity and polarization of the surface of MOF (001) so that the wave function is well distributed on the surface of MOF. Such a feature makes the ALC molecule interact well with the surface and allow proper charge transfer.

**Table 2:** The binding energy of ALC adsorbed on the Ag-MOF with the different (100), (010), and (001) surfaces.

Configuration	(1 0 0)	(0 1 0)	(0 0 1)
	*E*_*ads (eV)*_	*E*_*ads (eV)*_	*E*_*ads (eV)*_
A	− 0.15	− 0.157	− 0.654
B	− 0.12	− 0.145	− 0.599
C	− 0.09	− 0.131	− 0.577
D	− 0.07	− 0.122	− 0.544

Upon electrostatic interaction with the Ag-MOF through the thiosulfinate functional group (R-S-(O)-S-R), ALC forms a hydrogen bond with the substrate residues at a distance of 3.306 Å, which closely aligns with experimental results (3.4 Å) reported by Liu et al.^24^. Figure 2 illustrates the distances between the sulfur group of ALC molecule’s atoms and the surface atoms of the Ag-MOF (0 0 1) surface. The minimum energy configurations reveal different modes of interaction, primarily involving the C, S, and O atoms of ALC with the Ag and N atoms of the Ag-MOF structure. The E_bin_ values for configurations 0 1 0-A, 0 1 0-B, 0 1 0-C, and 0 1 0-D within the Ag-MOF system were found to be -0.157, -0.145, -0.131, and − 0.122 eV, respectively. In the Ag-MOF (1 0 0) system, these values were measured as -0.150 eV for 100-A, -0.120 eV for 100-B, -0.090 eV for 100-C, and − 0.070 eV for 100-D. This suggests a weak electrostatic interaction between the Ag-MOF and the ALC guest. This attractive interaction demonstrates how ALC molecules are retained within the pores of the MOF. The study finds that the (001) surface presents a greater number of accessible Ag metal sites, facilitating stronger physical adsorption. In contrast, the (100) and (010) surfaces are predominantly occupied by organic linkers, which result in comparatively weaker physisorptive interactions. Among the different configurations, Configuration D—presumably positioning the polar S = O group closest to the silver metal site—gains the most from both chemical interactions and vdW forces, resulting in the greatest reduction in its energy. Additionally, the BSSE correction addresses artificial stabilization arising from the use of finite basis sets. Its impact can vary across different configurations and might even lead to a change in their relative stability rankings if the basis set superposition error is not consistent. Shahabi and Raissi^25^ explored the interaction between 5-Fluorouracil (5-FU) and covalent organic frameworks. Their research revealed that 5-FU predominantly binds to these frameworks through physical adsorption, with adsorption energies ranging from − 0.15 to -0.354 eV. In our present study, the computed adsorption energies indicate that the ALC molecule can similarly undergo physical attachment to the surface of the Ag-MOF systems, specifically on the (0 1 0) and (1 0 0) crystal facets. Additionally, our Bader charge analysis reveals notable fluctuations in charge around the ALC guest molecule during its interaction with the Ag-MOF (0 0 1) surface^26^. This modification highlights the strong interaction between the metal site and the ALC guest. Additionally, all atoms surrounding the ALC guest exhibit charge changes in the presence of Ag-MOF (0 0 1). In configurations A and B, approximately 0.870 and 0.944 |e| of charges are transferred from ALC to the Ag-MOF (0 0 1) substrate, emphasizing the attractive interaction between the metal sites of the Ag-MOF and the ALC guest. Overall, our findings demonstrate the favorable adsorption behavior of ALC on Ag-MOF substrates, particularly with the Ag-MOF (0 0 1) configuration, which exhibits stable binding energies and strong interactions between the ALC molecule and the metal sites of the MOF. These results provide valuable insights into the potential of Ag-MOFs as effective carriers for ALC in biomedical applications. In Tables 3, 4 and 5, the E_bin_ related to ALC absorbed on the Ag-MOF surface and for different 001, 100 and 010 surfaces is displayed. The charge balance on sulfur and oxygen atoms and the total charge on the ALC molecule along with the electric dipole moment (DM) of the entire structure are displayed, for considering that the most stable states. Given that the 001-A to 001-D configurations represent the most stable structures for the ALC adsorption plane, it can be observed that the electric dipole moment in this arrangement is higher than the interactions between ALC and the MOF surfaces designated as (0 1 0) and (1 0 0). It is more than the interaction of ALC with other plane. Generally, the shape of the distributed charge of sulfur atoms is such that a positive charge is placed on them, and the oxygen charge is negative in all discussed states and about − 0.4e in the considered structures. For other planes interacting with ALC, is about − 0.2e. The charge on sulfur atoms is similar in all discussed states. It is well shown in Table 3 that the charge placed on the S1 atom varies between 0.55e to 0.65e for S1, and the charge placed on the S2 atom varies between positive 0.19e to 0.23e. The dipole moment of different configurations A to C in 001 state in the most stable state is between 70 and 72 Debye, while the dipole moment of the MOF in the pure state is about 42 Debye. This shows that the absorption of ALC has increased the dipole moment by about 30 Debye. The amount of this increase in other configurations is less and around 10 Debye. In general, the charge on the ALC molecule is negative, and the most charged state on the molecule is related to the stable structure of 001 integrating plane, which has a charge of about − 0.7e to -0.9e.

**Table 3:** The charge transfer (in electrons) and dipole moment (measured in Debye) characteristics of ALC adsorbed on the Ag-MOF with 001 surface.

Structure	Charge on S_1_	Charge on S_2_	Charge on O_7_	Charge on drug	DM
MOF (001)	–	–	–	–	42.24
A	0.625	0.198	− 0.403	− 0.870	72.77
B	0.640	0.208	− 0.406	− 0.944	72.37
C	0.651	0.197	− 0.406	− 0.730	72.01
D	0.551	0.236	− 0.411	− 0.741	70.11

**Table 4:** Charge transfer (in electrons) and dipole moment (measured in Debye) status of ALC adsorbed on the Ag-MOF with 100 surface.

Structure	Charge on S_1_	Charge on S_2_	Charge on O_7_	Charge on drug	DM
MOF (100)	–	–	–	–	19.08
A	0.246	0.138	− 0.218	− 0.153	29.31
B	0.280	0.188	− 0.210	− 0.194	29.27
C	0.213	0.135	− 0.209	− 0.127	27.16
D	0.201	0.128	− 0.213	− 0.118	28.01

**Table 5:** Charge transfer (in electrons) and dipole moment (measured in Debye) status of ALC adsorbed on the Ag-MOF with 010 surface.

Structure	Charge on S_1_	Charge on S_2_	Charge on O_7_	Charge on drug	DM
MOF (010)	–	–	–	–	19.38
A	0.201	0.167	− 0.265	− 0.172	23.11
B	0.209	0.164	− 0.223	− 0.146	23.48
C	0.214	0.163	− 0.274	− 0.176	21.76
D	0.207	0.158	− 0.218	− 0.197	22.39

**Fig. 4:**
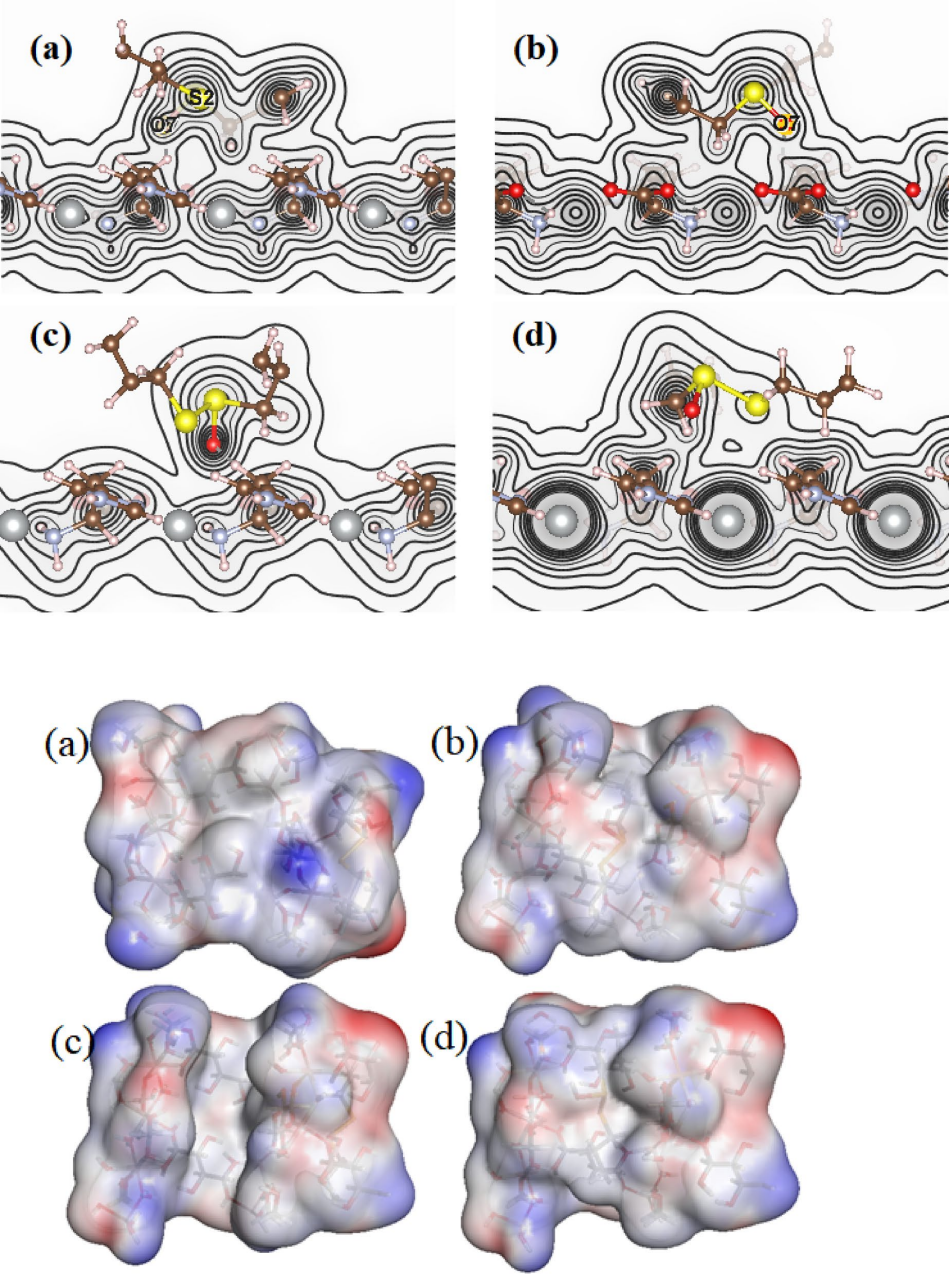
The MEP plots in contour and isosurface mode of ALC molecule adsorbed onto the Ag-MOF (0 0 1) surface for most stable.

The Molecular Electrostatic Potential (MEP) of ALC adsorbed onto the Ag-MOF (0 0 1) surface in its most stable configuration (D) was calculated using the DFT-D3 method. The results, presented in Fig. 4 in both contour and isosurface views, show distinct regions coloured in red, indicating sites favourable for the adsorption of negative charge. This distribution aligns with the inherent dipole moment of the ALC molecule. With the calculated charge on the adsorbed ALC molecule ranging from approximately − 0.6 |e| to -0.7 |e|, adsorption is highly likely to occur at these electropositive (red) regions. These findings further corroborate the strong interaction between ALC and the Ag-MOF substrate, suggesting efficient charge transfer and highlighting the potential of this system for applications in electronic and catalytic devices.

**Fig. 5:**
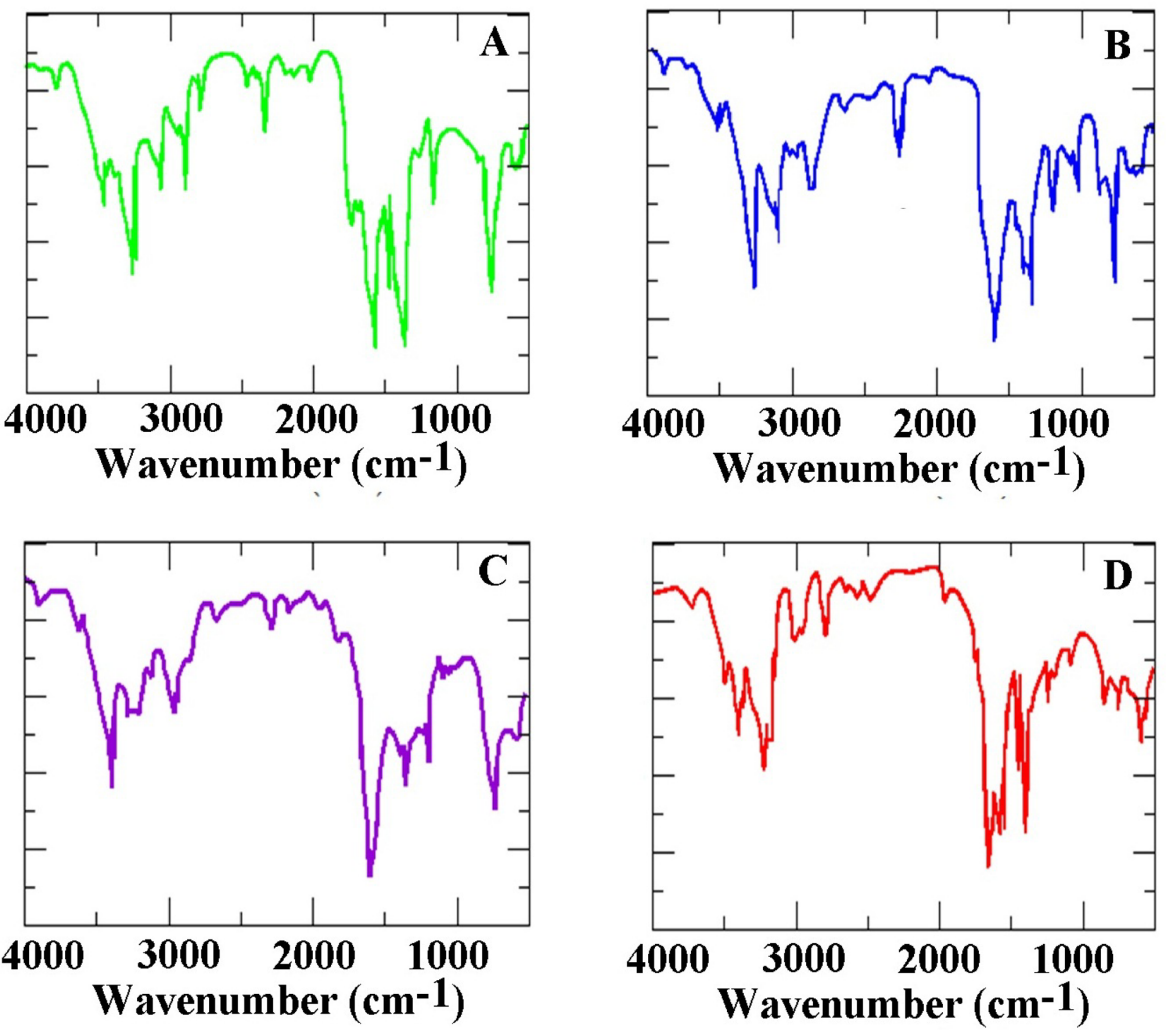
The infrared (IR) spectrum of ALC molecule loaded onto the Ag-MOF (0 0 1) surface.

The infrared (IR) spectra of ALC adsorbed on the Ag-MOF (0 0 1) surface in different configurations are illustrated in Fig. 5. The IR spectrum of the Ag-MOF (0 0 1) substrate displays prominent peaks at 400, 900, 1500, 2300, and 2500 cm^− 1^, which closely align with experimental results for this carrier^27,28^. These signals correspond to specific molecular vibrations within the Ag-MOF structure. Notably, a peak at 3698 cm^− 1^ indicates the presence of an O-H bond, while a signal at 1182 cm^− 1^ signifies a C-O bond (configuration 001-D). Additionally, vibrations associated with sp^3^-hybridized C-H and -CH_2_ bonds are observed at 3222, 1429, and 743 cm^− 1^. Peaks at 1696, 1525, and 934 cm^− 1^ are assigned to C = C, C-H, and = C-H double bonds, respectively. Vibrations at 1182 and 1048 cm^− 1^ can be attributed to the S = O bonds, while peaks at 667 and 514 cm^− 1^ indicate the presence of -S-S- and C-S bonds^29^. In Fig. 5, the vibrational modes associated with the adsorbed ALC molecule are illustrated at specific wavelengths: 313, 615, 665, 2885, and 2958 cm^− 1^. These modes represent the characteristic vibrational directions associated with the substituted groups on the ALC molecule. The analysis of these vibrational modes provides valuable insights into the structural changes and bonding interactions between ALC and the Ag-MOF (0 0 1) complex.

**Fig. 6:**
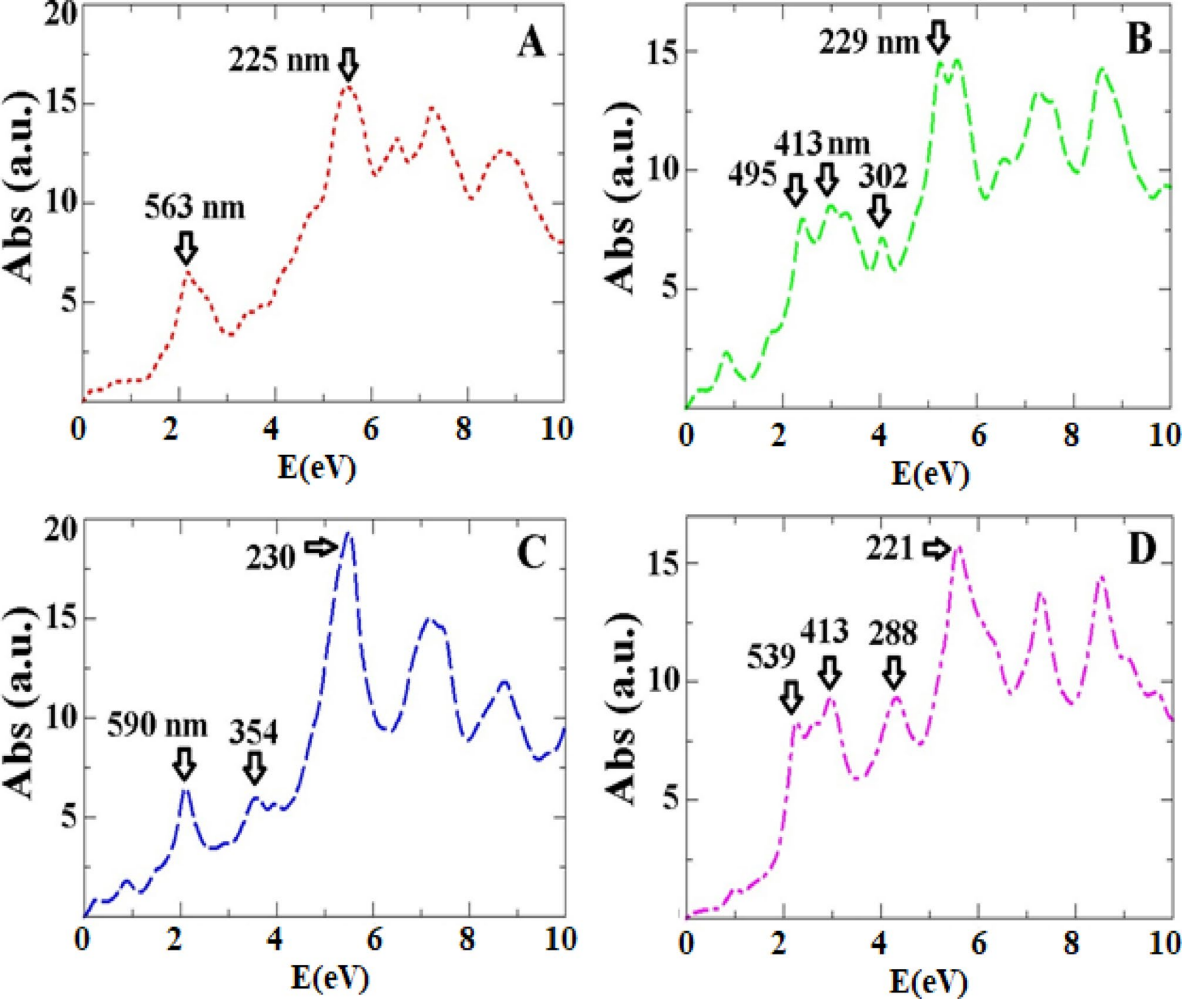
The optical absorption spectrum of ALC molecule loaded onto the Ag-MOF (0 0 1) surface.

Figure 6 presents the optical absorption spectrum for three more stable states of the ALC/Ag-MOF (0 0 1) complex. The spectrum reveals maximum peaks in the range of 1.5 eV (826 nm) to 6 eV (206 nm), which correspond to direct electron transitions from the valence band to the conduction band. These peaks are indicative of the absorption of light by the system. Lower-intensity peaks are observed in the energy range between 5.5 eV (225 nm) and 6.5 eV (190 nm), which are attributed to inter-band transitions resulting from the presence of the ALC molecule. The maximum absorption peaks for configurations 001-A, 001-B, 001-C, and 001-D are found to be 225, 229, 230, and 221 nm, respectively. Notably, the absorption peaks for different configurations exhibit an energy shift, particularly in the high-energy regions. This energy shift is primarily attributed to the alteration of narrow bands near the valence band, which are influenced by changes in the configuration of the adsorbed ALC molecule. These findings suggest that the electronic properties of the ALC/Ag-MOF (0 0 1) complex can be modulated by modifying the arrangement of the ALC molecule, leading to variations in the absorption characteristics of the system. Overall, the optical absorption spectrum provides valuable information about the electronic transitions and energy shifts in the ALC/Ag-MOF (0 0 1) complex, offering insights into its optical properties and potential applications in optoelectronic devices.

**Fig. 7:**
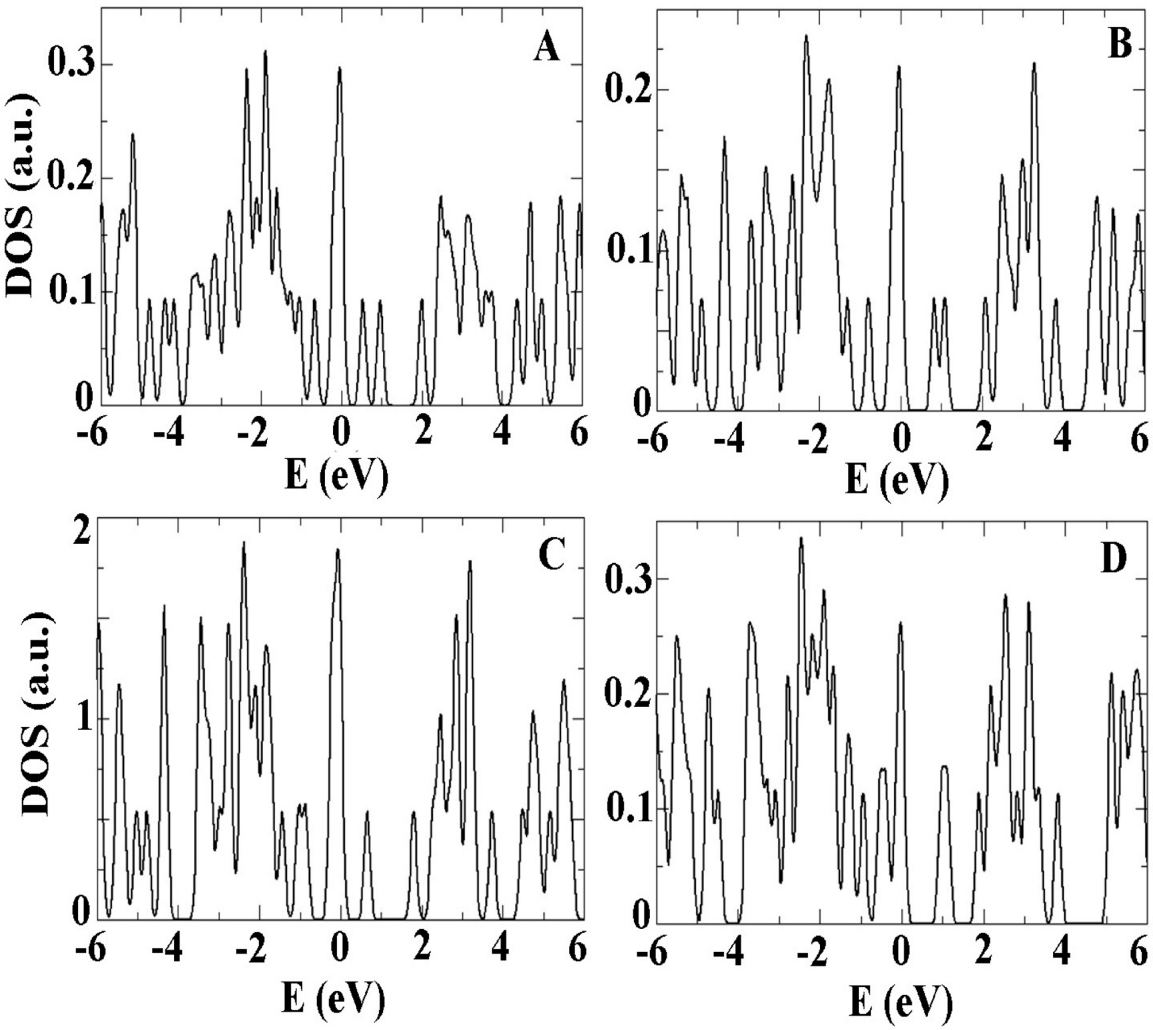
The DOS plots of ALC molecule loaded onto the Ag-MOF (0 0 1) surface.

We clarified that the UV-Vis spectra were generated using Time-Dependent Density Functional Theory (TDDFT) with the PBE exchange-correlation functional. Additionally, we have enhanced the discussion to clearly explain how the observed red-shift arises from the emergence of new electronic states introduced by ALC within the MOF’s band gap. These new states reduce the energy needed for electronic transitions, leading to the shift. Furthermore, the involvement of charge transfer excitations has been explicitly addressed. Figure 7 shows the density of states (DOS) for the four most stable configurations of the ALC/Ag-MOF (001) complex. Basically, the short wavelengths in the range of less than 2 electron volts in the absorption spectrum are caused by electron transitions from HOMO and LUMO levels, which are well shown in Fig. 7. In the range of 0 to 2 electron volts, there are two significant peaks that are LUMO + 1 and LUMO + 2 levels and contribute between 35% and 63% in creating small peaks in the same range of the absorption spectrum. We explain that the red-shift in the UV-Vis spectrum upon ALC adsorption is a direct consequence of new electronic states introduced by ALC within the MOF’s band gap. These new states reduce the energy required for electronic transitions, leading to the observed shift.

**Fig. 8:**
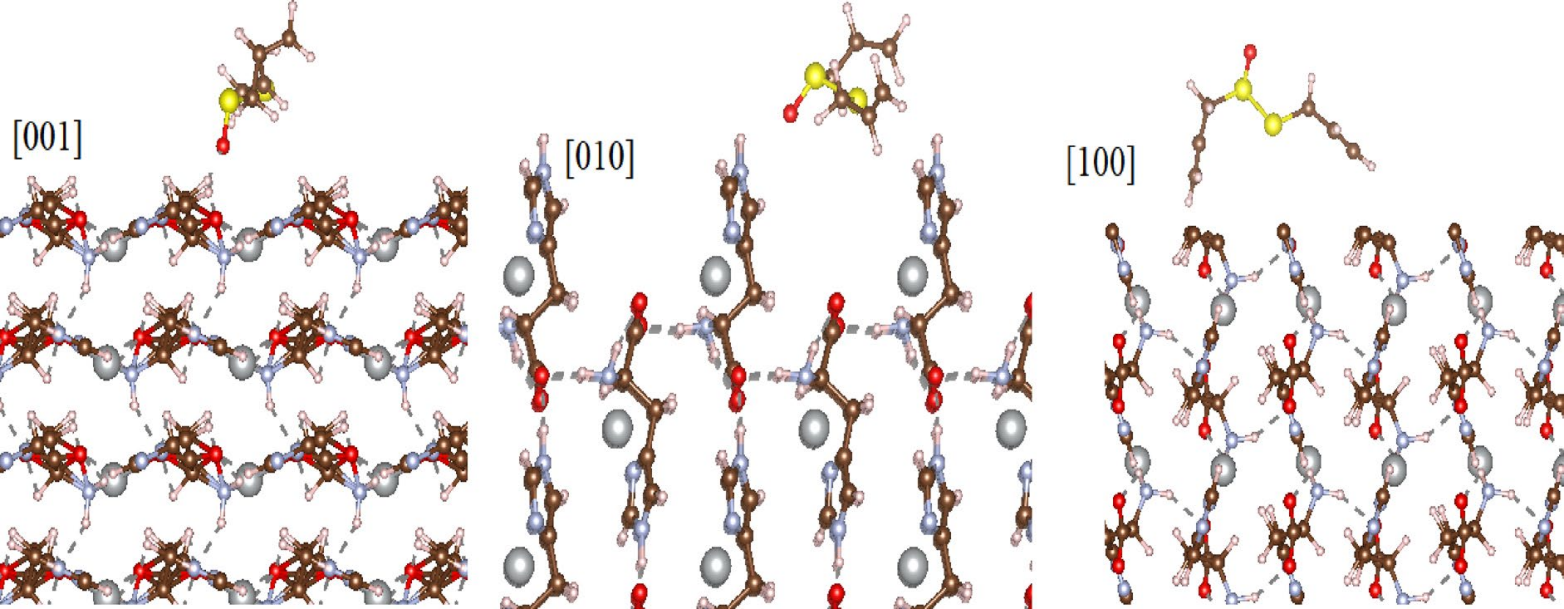
Structural configuration of ALC adsorbed onto the Ag-MOF (001), (010), and (1 0 0) surface in molecular dynamic study during 20 ps.

**Fig. 9:**
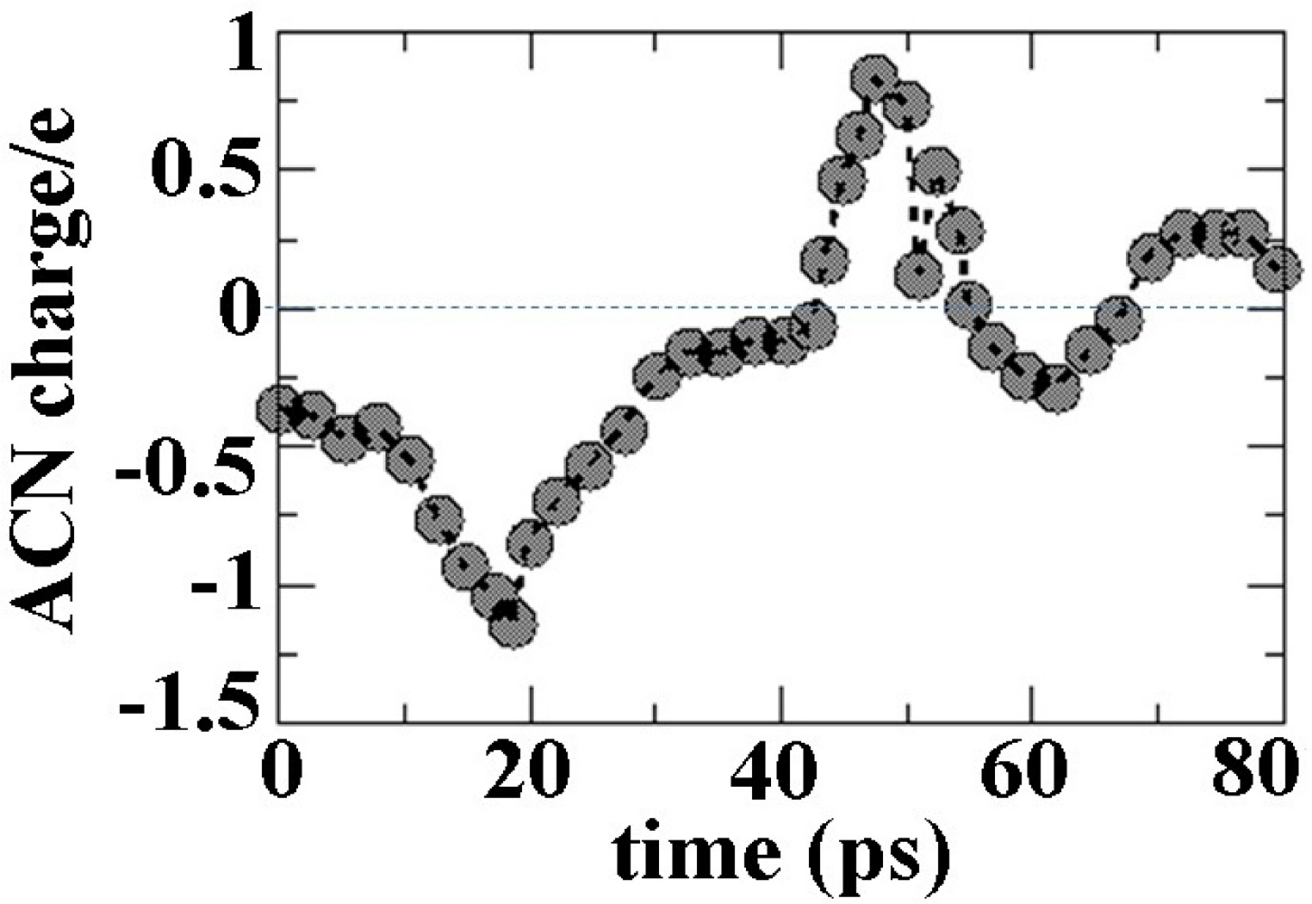
Charge changes of ALC adsorbed onto the Ag-MOF (0 0 1) surface during time evolution of adsorption.

Figure 8 shows the interaction process of the ALC molecule with the surface of 100 during a molecular dynamics study in the time frame of 20 to 80 ps. As it is clear in the figure, the porous space is in a suitable place for the absorption of the ALC molecule, and during 80 ps for the molecule interacted with the continuous surface (Fig. 8). The behavior of the charge transferred to ALC in this period of time is also shown in Fig. 9. The significant point is that in the range of 40 ps, the charge on the molecule has changed from negative to positive, and this factor is due to the intensification of the interaction of the ALC molecule with the MOF surface through S1 and S2 atoms.

**Fig. 10:**
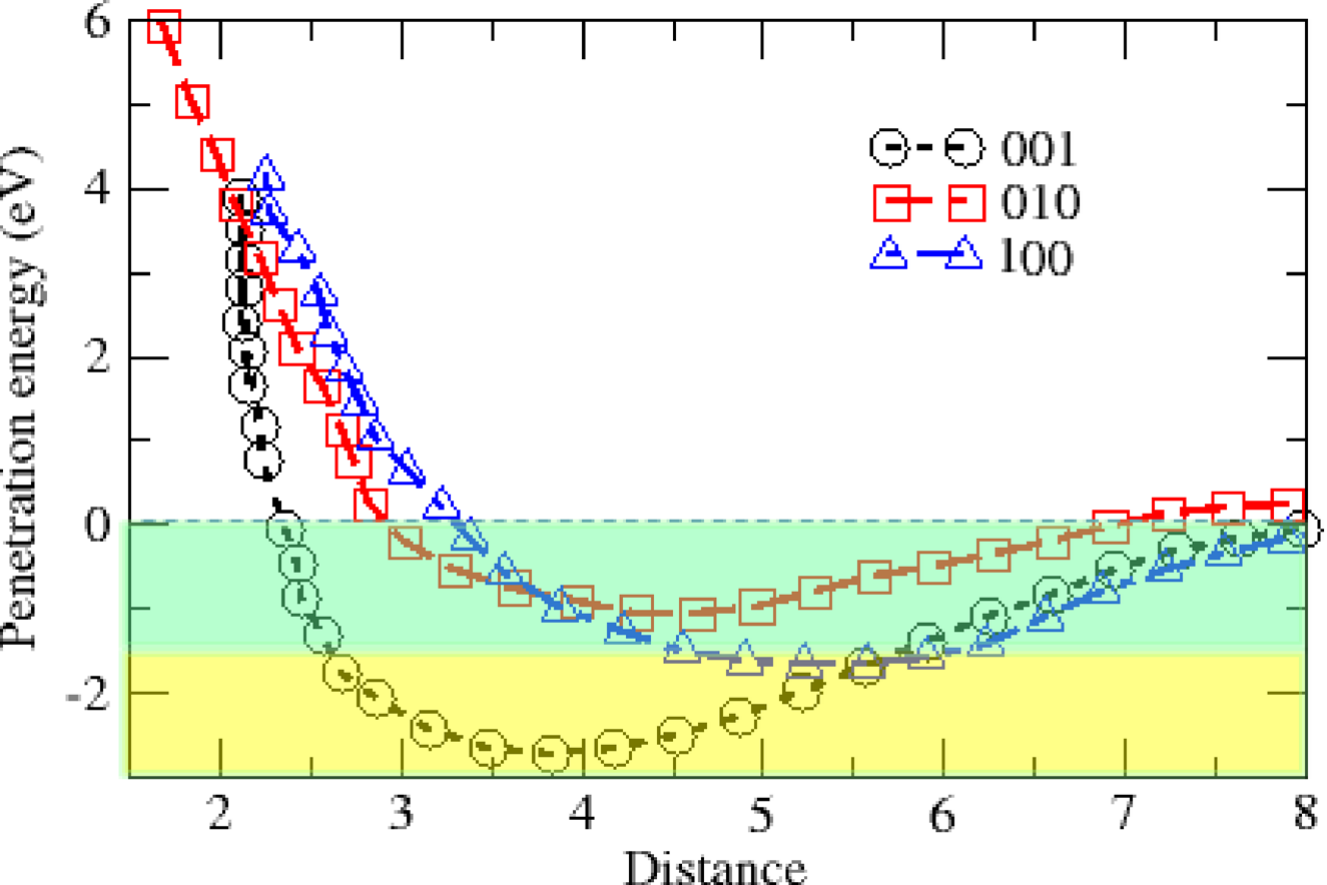
Penetration energy for ALC adsorbed on the (001), (010), and (100) surface of Ag-MOF.

The interaction of ALC molecule with the surface of MOF can create different alignments due to the effect of the surface potential in different directions. Figure 10 shows the energy required for the penetration of the ALC molecule into MOF in different crystal directions. Positive energy indicates repulsion and negative energy indicates attraction of molecules. As it is clear in the figure, the best direction for absorption, which has a significant difference compared to other directions, is the direction of 001. The green area shows physical absorption and the yellow area shows chemical absorption. According to the figure, the interaction of the ALC molecule with the porous surface of MOF in the direction of 100 and 010 is completely physical, but this interaction for the direction of 001 can be chemical in the range where the ALC molecule is located at a distance of 2.5 to 6 angstroms in a surface porous region.

**Fig. 11:**
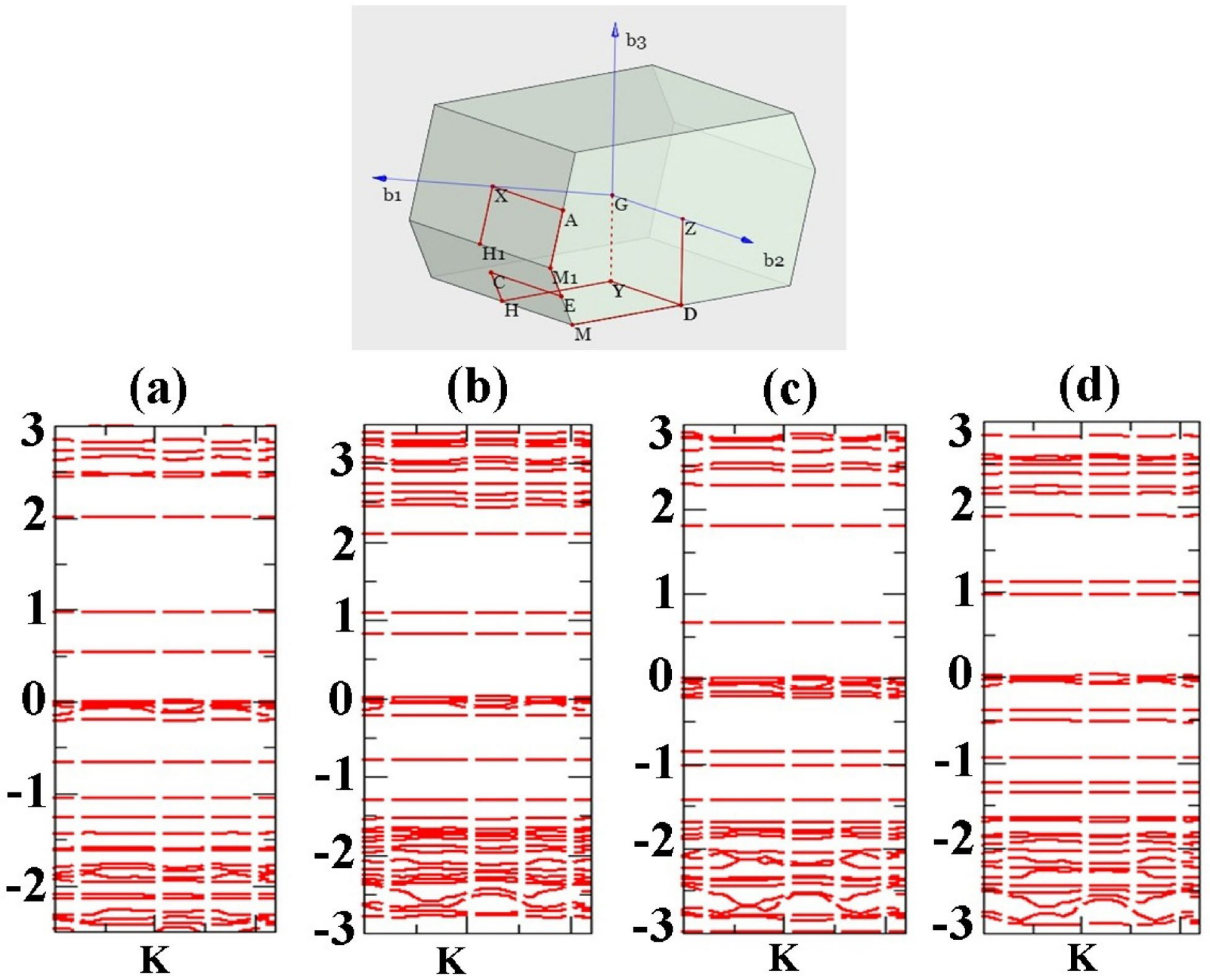
Band structure plot of ALC adsorbed onto the Ag-MOF (001) surface (a-d). The energy axis is measured in electron volts (eV), with key high-symmetry points indicated within the surface Brillouin zone.

For further study we have depicted band structure of most stable configurations along Can interacting with 001 Ag-MOF surface. Figure 11 presents the band structure diagram for the most stable states (a) to (d) of ALC adsorbed onto the Ag-MOF (0 0 1) surface. The first brilluon zone for considering high symmetry points also is shown in the figure. The narrow energy levels near the Fermi level and in the energy range of -0.5 eV represent the surface states that arise due to the adsorption of the ALC molecule on the surface. These surface states manifest as narrow and low-density levels, which do not exhibit a high density of states but can appear as intermediate levels in optical spectra or characterizations. The projected density of states (PDOS) on the ALC molecule adsorbed on the Ag-MOF (0 0 1) surface indicate that the energy levels near the band gap and below the Fermi level are localized on the ALC structure as consistent with the band structure diagram. These states primarily originate from the p orbitals of the ALC molecule and the π and π* orbitals resulting from hybridization with the surface states of the Ag-MOF (0 0 1) surface^30,31^. The analysis of the density of states provides insights into the electronic structure of the ALC/Ag-MOF (0 0 1) complex. The presence of surface states and the interaction between the ALC molecule and the Ag-MOF surface contribute to the formation of specific energy levels and hybridized orbitals, influencing the electronic properties and potential applications of the system in various optoelectronic and electronic devices.

## Conclusion

In summary, this study investigated the adsorption behavior of ALC molecules on the Ag-MOF system with different (1 0 0), (0 1 0), and (0 0 1) surfaces using DFT-D3 and MD simulations. Using the PBE-D3 functional, the binding energy (E_bin_) of ALC on the Ag-MOF (001) surface was found to be -0.894 eV in the 001-D configuration. This is more negative compared to -0.697 eV for configuration 001-A, -0.732 eV for configuration 001-B, and − 0.816 eV for configuration 001-C. The evaluation of binding energies reveals that the ALC in configuration D interacts more strongly with the Ag-MOF (0 0 1) surface, demonstrating a higher binding energy indicative of robust physical adsorption. In contrast, the interactions with the Ag-MOF (0 1 0) and Ag-MOF (1 0 0) surfaces are comparatively weaker, corresponding to a milder physisorption process. The changes in dipole moment for the Ag-MOF surface increased from 42.24 Debye to 72.77 (001-A), 72.37 (001-B), 72.01 (001-C), and 70.11 (001-D) Debye, suggesting enhanced solubility. The UV-Vis spectrum demonstrated the maximum absorption peaks in the region of 225 (001-A), 229 (001-B), 230 (001-C), and 221 (001-D) nm compared to the perfect substrate (217 nm), with significant intensity variations depending on the different configuration interactions of ALC with the MOF backbone. The computed IR spectra of ALC and Ag-MOF (0 0 1) were in agreement with experimental measurements, validating the accuracy of the theoretical predictions and supporting the reliability of the DFT calculations. Bader Charge Analysis (BCA) examined the state of charge transfer between the MOF and ALC, revealing its dependence on the structural properties of the complex. The electronic structure of the Ag-MOF was found to undergo alterations upon ALC adsorption, as demonstrated by the theoretical analysis. These findings offer valuable insights into the properties of ALC when adsorbed onto Ag-MOFs, providing a foundation for further exploration of their role in biomedical applications.

## Data Availability

All data generated or analysed during this study are included in this published article.
